# Supplementary material for: Study on the sustainability of ancient canal towns on the basis of the topological structure analysis of streets and lanes: A case study of the Xixing ancient town
Source: PLoS One. 2023 Jan 6;18(1):e0279979. doi: 10.1371/journal.pone.0279979 (PMC9821790; doi:10.1371/journal.pone.0279979)
Supplement: S1 File — (DOCX) [file pone.0279979.s001.docx]

**The statement and keywords**

This analysis is conducted by the method of space syntax aiming to calculate the complex relationship from all the streets to all the others. The integration measures how far a street is connected to all the other streets within a given radius; and meanwhile, the choice calculates the possibility of the shortest paths going through a given street that lies on those shortest paths. All the analyses were conducted by Depthmap.

Keywords: complex relationship; integration measures; choice; Depthmap
