# Supplementary material for: Study on the sustainability of ancient canal towns on the basis of the topological structure analysis of streets and lanes: A case study of the Xixing ancient town
Source: PLoS One. 2023 Jan 6;18(1):e0279979. doi: 10.1371/journal.pone.0279979 (PMC9821790; doi:10.1371/journal.pone.0279979)
Supplement: Attachment — Submitted filename: Response Letter.doc [file pone.0279979.s005.doc]

Dear Editor,

Thank you very much for your decision letter and suggestions on our manuscript (PONE-D-21-07427) entitled “Study on the Sustainable Renewal Strategy of Ancient Towns with Canals on the Basis of the Topological Structure Analysis of Streets and Lanes: A Case Study of the Xixing Ancient Town”. Accordingly, we have revised the manuscript. In addition, point-by-point responses to the suggestions are listed below this letter.

We hope that the revision is acceptable for the publication in your journal.

Look forward to hearing from you soon.

With best wishes,

Yours sincerely,

Ning Wang

**Replies to Suggestions**

Suggestion 1: Introduction: No application literature

Response: In the introduction part, we supplemented the basic information about the Grand Canal, briefly described the difficulties faced by the ancient towns with canal in the process of urbanization, and introduced the research perspective and content. Besides, we emphasized the study should not be limited to the ancient town itself, but also explore the relationship between the ancient town and the urban system from different spatial scales, which is also the innovation of this study.

Suggestion 2: Discussion: It did not explain the significance or contribution of his own research and the research of domestic and foreign scholars, nor did it explain the limitations of the research.

Response: We re-organized the literature review, and elaborated from three perspectives: Street spatial form, street spatial behavior and street spatial structure. Based on this, we analyzed the current research trend, and also pointed out that most of the existing studies on the ancient towns are oriented to the internal traffic system of a single town, and few consider the research object as a part of the city itself, which lacks objectivity.

Suggestion 3. References: There are only 17 references and all of them are Chinese.

Response: We have expanded the references to 26. Half of them are Chinese.
