# Supplementary material for: Study on the sustainability of ancient canal towns on the basis of the topological structure analysis of streets and lanes: A case study of the Xixing ancient town
Source: PLoS One. 2023 Jan 6;18(1):e0279979. doi: 10.1371/journal.pone.0279979 (PMC9821790; doi:10.1371/journal.pone.0279979)
Supplement: Attachment — Submitted filename: PONE-D-22-18649-review_comments.docx [file pone.0279979.s006.docx]

Dear Editor,

Thank you very much for offering me the opportunity to review this manuscript. The manuscript deals with an interesting research topic, having a well organized but poorly addressed research study. Therefore, there is room for significant methodological, morphological, and argumentation improvements to be made, prior it to be accepted for publication at the PLOS ONE journal. To this end my review comments can be considered.

Thank you very much again. I am always at your reviewing disposal.

Kind regards,

Full name of reviewer

**Review comments for the manuscript: PONE-D-22-18649**

*Study on the Sustainable Renewal Strategy of Ancient Towns with Canals on the Basis of the Topological Structure Analysis of Streets and Lanes: A Case Study of the Xixing Ancient Town*

The manuscript deals with an interesting research topic, having a well organized but poorly addressed research study. Therefore, there is room for significant methodological, morphological, and argumentation improvements to be made, prior it to be accepted for publication at the PLOS ONE journal. To this end the following review comments can be considered.

1) A rough estimation of chronological flash-back to the century of the Xixing ancient town’ foundation, it can be provided in the Abstract section (in approximate century B.C.).
2) In case of some input data or results derived from the analysis, then, a selected and representative portion of them they can be collectively denoted in Abstract section, enabling a more precise representation of the relevant analysis and findings in the Abstract extract, accordingly.
3) At the Introduction section authors are recommended to reorganize the relevant theoretical coverage into two distinct and separated subsections, relevant to two subsections/subheadings of: a) Geographical overview of ancient towns in China and b) Chronological overview of ancients towns establishment and evolution to the today Chinese context. The spatial characteristics approached in section 2. Literature Review are well organized. However, a missing information of both narratives of sections Introduction, Literature Review is that there is absolutely missing numerical information-outcomes that could be collectively represented in the form of a Table at the end of section 2. There is not need authors to recall the narrative statements, but to enrich them with numerical information fitting to the already, or to-be-developed, subsections of main sections 1 and 2. These data of 8-10 citations are adequate.
4) At the Methodology section authors are recommended to briefly represent the land uses of the ancient town examined, in terms of: a) agricultural zone and area occupied (in km^2, or as percentage of the total area of the town), urban zones: pedestrian, streets and lanes (in km^2, or as percentage of the total area of the town), irrigation system and rainfall profile (in mm), domesticated area.
5) In my opinion the heading/section of “Research Design” it is part of the Methodology section, thus, it can be transferred in it, accordingly. Besides, the main frame of headings should be only that of (adding those main headings, where missing): 1. Introduction, 2. Literature Review, 3. Methodology, 4. Results, 5. Discussion, 6. Conclusions and Future Research Orientations. Then, other main headings can be degraded to subheadings’ relocated into the aforementioned headings-frame. Moreover, starting from the 1. Introduction and end to 6. Conclusions and Future Research Orientations section, all embodied subheadings can be titled as: 1.1, 1.2, ……., 2.1, 2.2, …..3.1, 3.2, ….., 4.1, ….4.2, ……., in successive order and numbering, not to be plenary given.
6) At the Discussion section should be reorganized focusing on the today reflections and commercial commitments/services offered, in alignment with the stated research objective: “spatial data such as the number of commercial entities on each street segment, the area, area width, depth of single building and spatial syntax parameters is carried out”. To this end the following key-determinants can be briefly outlined: a) visiting the ancient towns in China, as an inseparable part of the today tourism industry, b) commercial, manufacturing and industrial activities and products/goods’ offered, c) the role of technology in the main community uses: agricultural sector, irrigation technologies, manufacturing and public services accommodated, d) role of governmental policies and motivations scheduled in supporting the ancient towns heritage, protection, and development of related to infrastructure at the nearby, to ancient towns, areas in China. To this end the “Further Discussion”:

-It can be detached, “upgraded as main section”, and presented as separated “Discussion” section, since it contains purely argumentative statements, though all of them have to be accompanied by cross-citations that are currently missing.

-It can be accompanied by numerical information, data, quantitative results, enabling a more precise and comprehensive “guidance” of research outcomes, proposals, and recommendations to areas and contexts of the same research- and spatial- characteristics to be derived and applied.

7) Since Figures and all subfigures of 2 up to 6 are graphically liking to each other, it is highly recommended authors to either provide a more detailed information/interpretation of the comparing and contrasting them, revealing what are the distinct characteristics from the one subfigure to the other one. To this end authors can update these graphs by adding indicators of presentation in the form of arrows into these maps, it could be highly supportive and recommended.
8) There is a long arrangement of numbers of 6 or more decimal digits and fluctuated precision, like that of lines 205, 217, 229-230, but also in Tables data. Therefore, revising and fixing the precision of all data to only 3 decimal digits it is highly recommended for the scopes of this manuscript.
9) Figure 7 can be presented in a white-coloured background, in better visual contrast with the trend-line dots to be achieved.
10) Literature enrichment with more and relevant published papers it is recommended, thus, checking and citing among the below listed studies, where missing and where matching to the revised manuscript, it can be undertaken.

Scopus

EXPORT DATE:12 Jul 2022

Wang, Z., Zhang, H., Yang, X., Li, G.

36469470000;57373042100;57373940300;57373581900;

Neighborhood streets as places of older adults’ active travel and social interaction – A study in Daokou ancient town

(2022) Journal of Transport and Health, 24, art. no. 101309, . Cited 1 time.

https://www.scopus.com/inward/record.uri?eid=2-s2.0-85121263625&doi=10.1016%2fj.jth.2021.101309&partnerID=40&md5=708e7f95f3c811844c51f5b25830e74f

DOI: 10.1016/j.jth.2021.101309

AFFILIATIONS: Department of Architecture, Henan University, Kaifeng, China;

Department of Architecture, Henan University of Technology, Zhengzhou, China;

Department of Architecture, Zhengzhou University, Zhengzhou, China

ABSTRACT: Introduction: Neighborhood streets are convenient places for older adults to engage in behaviors for active living, such as walking (active travel) and chatting with neighbors (social interaction). Street environments and older adults' active living in ancient towns need investigation. Taking Daokou ancient town in China as an example, this research observed older adults’ active travel and social interaction on two neighborhood streets and investigated the difference in social engagement between older-adult groups on different streets. Methods: On-site non-participant observation was conducted for four weekdays with seven 30-min sections per day. Data of 350 older adults’ active travel and social interaction on these streets were collected. Street environmental factors were measured and classified into four categories in terms of active-travel promotion: typology, motivators, functionality, and safety. To identify the differences in social engagement between the groups by street, one-way ANOVA tests were conducted after controlling for a significant confounding variable (daypart). Results: Among the older adults, the most popular type of active travel was independent walking (67%). Of their social interaction, the most popular types were staying and chatting (61%), group walking, and chess or card playing. On the street considered more age-friendly to active travel, older adults engaged in more social interaction in the mid-mornings and afternoons (p < 0.05). Conclusion: This study highlighted older adults' active living on neighborhood streets in ancient towns. The findings can be used to create street affordances for older adults’ active travel and social interaction, and produce healthy outcomes through the refinement of design and transportation policies and practice on street intervention. © 2021 Elsevier Ltd

AUTHOR KEYWORDS: Active living; Aging; Environment; Health; Historic

DOCUMENT TYPE: Article

PUBLICATION STAGE: Final

SOURCE: Scopus

Manakina, A., Nikolaeva, V.

56134235500;57218826705;

Formation of sustainable development of bicycle and pedestrian zones in a modern city

(2020) IOP Conference Series: Materials Science and Engineering, 890 (1), art. no. 012179, .

https://www.scopus.com/inward/record.uri?eid=2-s2.0-85090348649&doi=10.1088%2f1757-899X%2f890%2f1%2f012179&partnerID=40&md5=c0bf49b51025943e083d8eaf6c93b826

DOI: 10.1088/1757-899X/890/1/012179

AFFILIATIONS: Industrial University, Tyumen, 625000, Russian Federation

ABSTRACT: The article is devoted to the current theme of bicycle and pedestrian space development in the developing city, as well as to attracting residents to the landscaped parts of the city for active recreation. The authors consider various types of activity, the implementation of which requires landscaped and safe areas, as well as enumerate the positive aspects associated with the introduction of active recreation in the daily life of residents, therefore, improve the quality of life and increase investment attractiveness of the region. The aim of the study is to substantiate the significance for the city of Tyumen of the creation and combination of the cycling and pedestrian zones, which shall contribute to the welfare in the sphere of the active recreation of citizens. The methods of the study are the following: analysis of the population dynamics in the city of Tyumen, which affects the socio-economic development of the region; analysis of the structure of the central part of the city of Tyumen, indicating the lack of greenery; consideration of the option of bicycle lane placement near the street Dzerzhinsky city of Tyumen. © Published under licence by IOP Publishing Ltd.

AUTHOR KEYWORDS: cycle paths; cycling; ecology; gardening; pedestrian traffic; transport infrastructure

DOCUMENT TYPE: Conference Paper

PUBLICATION STAGE: Final

SOURCE: Scopus

Yu, X., Ma, S., Cheng, K., Kyriakopoulos, G.L.

53865793000;57218326388;57218326647;6603382498;

An evaluation system for sustainable urban space development based in green urbanism principles-a case study based on the Qin-Ba mountain area in China

(2020) Sustainability (Switzerland), 12 (14), art. no. 5703, . Cited 24 times.

https://www.scopus.com/inward/record.uri?eid=2-s2.0-85088828018&doi=10.3390%2fsu12145703&partnerID=40&md5=f4c407b3b707d7b77b1350577b4e98e5

DOI: 10.3390/su12145703

AFFILIATIONS: School of Architecture, Chang'an University, Xi'an, 710061, China;

Electric Power Division, Photometry Laboratory, School of Electrical and Computer Engineering, National Technical University of Athens, Athens, 15780, Greece

ABSTRACT: Since the 20th century, the deterioration of the ecological environment around the world has challenged urban space construction. With the development of urbanization, the consumption of resources and energy has increased, the level of biodiversity has decreased, environmental pollution is approaching the critical level, and the contradiction between human habitat activity and ecological environment has become increasingly prominent. The sustainable development of urban space along with its economic and social benefits, taking into account the quality of life and ecological environment, has become a new and important subject that needs to be explored. In this study, the indices of the evaluation system for sustainable urban spatial development in regions with underdeveloped economies but rich in ecological resources are arranged in sequence through the systematic coupling analysis of collaborative evaluation information and a quantitative analysis. The influences of urban space elements on sustainable urban development are disclosed. On the basis of the generated data, an evaluation system for sustainable urban spatial development with a complete set of information is proposed. The proposed system is applicable to urban spatial development evaluation in regions in China with underdeveloped economies but rich in ecological capital. First, the basic concept of system coupling is introduced, and a coupling relationship between urban sustainable development and urban space is proposed. Second, the elements of urban space and the sustainable development in the Qin-Ba mountain area are extracted, and the precedence diagram method is used to construct a sustainable evaluation system for urban space development in the Qin-Ba mountain area. Third, the sustainable evaluation process of urban spatial development is proposed. Finally, the sustainable evaluation system for urban spatial development in the Qin-Ba mountain area is applied to evaluate the urban spatial development in Shangluo, Qin-Ling Mountains, China. The results show that, among the investigated 14 indicators, the proportion of industrial land use mainly influences sustainable urban spatial development. As for the rest of the index factors, per capita green land area and green coverage ratio of built-up areas, per capita urban construction land area, proportion of forestry area, greening rate of built-up areas, total industrial dust emission density, proportion of cultivated area, and average volume fraction of residential areas are the secondary influencing factors of sustainable urban spatial development. The evaluation system in this research is constructed with the three aspects of "green coordination", "green development", and "green sustainability" of sustainable urban spatial development, and it complements the evaluation contents of urban-rural ecological space coordination, land resource protection, and green development community, and so on. The conclusion of this study not only can provide a useful reference for urban spatial development planning for underdeveloped ecological capital areas of China but also can provide a theoretical basis for the management and control policy of sustainable urban spatial development. © 2020 by the authors.

AUTHOR KEYWORDS: City spatial development; Green coordination; Green development; Green sustainability; Sustainable evaluation system

DOCUMENT TYPE: Article

PUBLICATION STAGE: Final

SOURCE: Scopus

Nomura, R., Shan, S., Mori, S.

57191923705;57204313367;37112638600;

Analyzing spatial structure of traditional houses in old towns with tourism development and its transformation toward sustainable development of residential environments in hexia old town, in China

(2018) Sustainability (Switzerland), 10 (10), art. no. 3809, . Cited 6 times.

https://www.scopus.com/inward/record.uri?eid=2-s2.0-85055187483&doi=10.3390%2fsu10103809&partnerID=40&md5=69acac765079e477ed50bff363e6ac4c

DOI: 10.3390/su10103809

AFFILIATIONS: Faculty of Engineering, Hokkaido University, Sapporo, 060-8628, Japan;

Shanghai Urban Construction Design and Research Institute (Group) Co., Ltd., Shanghai, 200125, China

ABSTRACT: This study elucidates the spatial structure of traditional houses, and its transformation toward sustainable development of residential environments in old towns that are currently the focus of tourism development initiatives. Hexia old town in Huai'an District, China, was chosen for this study involving a field survey, questionnaire survey, and interviews. Data analysis identified three periods of residential transformation and three patterns of the residential transformation process. In all cases, there was low awareness of building conservation measures and lack of tourism development by the government, which has translated into ongoing residential transformations according to the demands of daily life. However, in the interests of tourism development, some businesses have started making use of vacant residences and traditional homes, and the relative proximity to work and home imply sustainability of residents' lifestyles under these conditions. An improvement plan for an old town requires a thorough understanding of the circumstances surrounding residential modifications executed by residents, the problems faced in the living environment, as well as efforts to increase the residents' awareness of the issue of sustainable living in that old town. © 2018 by the authors.

AUTHOR KEYWORDS: China; Living environment; Spatial structure; Tourism development; Traditional house

DOCUMENT TYPE: Article

PUBLICATION STAGE: Final

SOURCE: Scopus

Dičiunaite-Rauktiene, R., Gurskiene, V., Burinskiene, M., Maliene, V.

57201215996;8963424300;10040854900;24332653600;

The usage and perception of pedestrian zones in Lithuanian Cities: Multiple Criteria and comparative analysis

(2018) Sustainability (Switzerland), 10 (3), art. no. 818, . Cited 9 times.

https://www.scopus.com/inward/record.uri?eid=2-s2.0-85044007619&doi=10.3390%2fsu10030818&partnerID=40&md5=e9d368d976fc11b7515f76720018ed38

DOI: 10.3390/su10030818

AFFILIATIONS: Institute of Land Management and Geomatics, Faculty ofWater and Land Management, Aleksandras Stulginskis University, Studentu 11, Akademija, Kaunas distr., LT-53361, Lithuania;

Road Department, Faculty of Environmental Engineering, Vilnius Gediminas Technical University, Sauletekio ave. 11, Vilnius, LT-10223, Lithuania;

Department of the Built Environment, The Built Environment and Sustainable Technologies Research Institute, Faculty of Engineering and Technology, Liverpool John Moores University, Byrom street, Liverpool, L3 3AF, United Kingdom

ABSTRACT: As pedestrian zones are public spaces in urban areas, they are important in terms of meeting people's needs. However, it is worth noting that attention should be paid not only to the development of the physical infrastructure, but also to a sustainable balance between the socio-economic and environmental aspects. To guide urban planning and management initiatives towards more sustainable patterns, it is essential to re-examine the already existing characteristics of cities, establishing how they are used and perceived by inhabitants. The present research suggests environmental, economic and social criteria that determine a greater vitality of pedestrian zones in cities and better life quality for the inhabitants. A questionnaire survey was used to assess common attitudes regarding the research topic in major cities in Lithuania. The multiple criteria decision-making COPRAS (COmplex PRoportional ASsessment) method was used for the formation of a priority queue. The research results showed the attitudes of inhabitants towards pedestrian zones in Vilnius, Kaunas and Klaipeda. The inhabitants of these three cities regarded social and environmental criteria groups as the most significant. Contrary to what was expected and anticipated, respondents do not consider economic criteria as playing a key role in the sustainable preservation and development of pedestrian zones. © 2018 by the authors.

AUTHOR KEYWORDS: Assessment of pedestrian zones in Lithuania; Criteria for successful pedestrian zones; Multiple criteria analysis; Pedestrian zones in cities; Sustainability of pedestrian zones

DOCUMENT TYPE: Article

PUBLICATION STAGE: Final

SOURCE: Scopus

Yang, F.

36607241700;

Investigating wintertime pedestrian wind environment and user perception in dense residential neighbourhood in a city of hot-summer and cold-winter climate zone, China

(2017) Indoor and Built Environment, 26 (3), pp. 392-408. Cited 3 times.

https://www.scopus.com/inward/record.uri?eid=2-s2.0-85018181386&doi=10.1177%2f1420326X15620257&partnerID=40&md5=3afd66a42594b9f0e83e188c3d22fbdc

DOI: 10.1177/1420326X15620257

AFFILIATIONS: College of Architecture and Urban Planning (CAUP), Tongji University, 1239 Siping Road, Shanghai, 200092, China;

Key Laboratory of Ecology and Energy-Saving Study of Dense Habitat, Tongji University, Ministry of Education, Shanghai, China

ABSTRACT: Urban wind environment could have an impact on pedestrian's comfort and safety, as well as pollution dispersion and building energy consumption. For cities in the hot-summer and cold-winter climate zone of China, a proper design residential neighbourhoods is important to facilitate urban ventilation in hot and transient seasons and to protect users from strong winds in cold season. This paper reports the results of field measurements and a questionnaire survey in a large residential development with three different types of housings. Micrometeorology measurement was carried out at the pedestrian height level as well as at a rooftop reference station. Pedestrians' subjective perception on wind and thermal comfort was recorded through a guided interview and questionnaire survey during the measurement. The measured wind velocity ratio is highest in the long-linear high-rise building layout, and is the lowest in the mid-rise linear building layout. Eight-seven per cent of respondents felt fairly comfortable living in the long-linear high-rise building layout, only 7% less than the mid-rise building layout. For similar housing forms in Shanghai, the wintertime wind shelter may not be critical compared with summertime ventilation requirement, and that the site planning and housing design should focus mainly on summertime wind channelling. © The Author(s) 2015.

AUTHOR KEYWORDS: Hot-summer cold-winter; Housing form; Pedestrian comfort; Urban design; Urban ventilation; Wind perception

DOCUMENT TYPE: Article

PUBLICATION STAGE: Final

SOURCE: Scopus

Guo, W., Meng, X., Zhang, Y., Wang, N.

57199262830;57194581397;57194584688;57194582523;

Spatial development model of sustainable tourism town based on smart city

(2017) Agro Food Industry Hi-Tech, 28 (1), pp. 853-857.

https://www.scopus.com/inward/record.uri?eid=2-s2.0-85020975732&partnerID=40&md5=01cbd36b08a8a0f2ca20ec0ff56d3c2e

AFFILIATIONS: School of Economics and Management, Yanshan University, Institute of Modern Tourism Industry and Organization Development, Yanshan University, Qinhuangdao, Hebei, China;

School of Economics and Management, Yanshan University, Qinhuangdao, Hebei, China

ABSTRACT: With the strong support of the rapid development of Internet. "Intelligent" and "Wisdom" has become an important direction for the development of various industries. Smart city integrates the policy design and practice innovation in the three areas of urbanization, industrialization and information technology, which is the focus of China's new round of economic development. Therefore, the spatial development model of sustainable tourism town based on smart city was studied in this paper. On the basis of brief introduction of relevant theories and concepts, the sustainable development model is established based on practical demonstration to discuss the future spatial development and planning model of tourism towns. The results show that the concept of sustainable development wisdom tourism towns is very necessary, and the establishment of spatial development model is the most important.

AUTHOR KEYWORDS: Smart city; Space development; Sustainable; Tourism town

DOCUMENT TYPE: Article

PUBLICATION STAGE: Final

SOURCE: Scopus
