# Supplementary material for: Study on the sustainability of ancient canal towns on the basis of the topological structure analysis of streets and lanes: A case study of the Xixing ancient town
Source: PLoS One. 2023 Jan 6;18(1):e0279979. doi: 10.1371/journal.pone.0279979 (PMC9821790; doi:10.1371/journal.pone.0279979)
Supplement: Attachment — Submitted filename: PONE-D-22-18649R1-review_comments.docx [file pone.0279979.s008.docx]

**Review comments for the revised manuscript: PONE-D-22-18649R1**

*Study on the Sustainable Renewal Strategy of Ancient Towns with Canals on the Basis of the Topological Structure Analysis of Streets and Lanes: A Case Study of the Xixing Ancient Town*

Dear Editor,

Thank you very much for offering me the opportunity to review this revised manuscript. At this revised manuscript authors developed a thorough and substantial reorganization of their initial manuscript, having the review comments addressed in a meticulous manner. The narrative flow is smooth, the methodology analysis is sound, and the research outcomes are insightful. In this respect the revised manuscript sustains novel features of regional and wider socio-cultural impact on the Chinese context, thus, it can be accepted for publication at the PLOS One journal as is.

Thank you very much again. I am always at your reviewing disposal.

Kind regards,

Dr. Grigorios L. Kyriakopoulos
