# Supplementary material for: Study on the sustainability of ancient canal towns on the basis of the topological structure analysis of streets and lanes: A case study of the Xixing ancient town
Source: PLoS One. 2023 Jan 6;18(1):e0279979. doi: 10.1371/journal.pone.0279979 (PMC9821790; doi:10.1371/journal.pone.0279979)
Supplement: Attachment — Submitted filename: Response to Reviewers.docx [file pone.0279979.s009.docx]

Dear Editor,

We are truly grateful to yours and other reviewers’ critical comments and thoughtful suggestions on our manuscript (Basis of the Topological Structure Analysis of Streets and Lanes: A Case Study of the Xixing Ancient Town).

Based on these comments and suggestions, we have made careful modifications on the original manuscript. All changes made to the text are in yellow color. We hope the new manuscript will meet your journal’s standard. Below you will find our point-by-point responses to the reviewers’ comments/questions.

We hope that these revisions are satisfactory and that the revised version will be acceptable for publication in PLOS ONE journal.

Thank you very much for your work concerning our paper. Wish you all the best!

**Review comments for the manuscript: PONE-D-22-18649**

*Study on the Sustainable Renewal Strategy of Ancient Towns with Canals on the Basis of the Topological Structure Analysis of Streets and Lanes: A Case Study of the Xixing Ancient Town*

Thank you for submitting your manuscript to PLOS ONE. After careful consideration, we feel that it has merit but does not fully meet PLOS ONE’s publication criteria as it currently stands. Therefore, we invite you to submit a revised version of the manuscript that addresses the points raised during the review process.

1. Authors(s) already provided the abstract’s content (the purpose/background, method used, results). However, please provide clear methods on the studies that have been conducted. Please make it short and sharp. In addition, I recommend writing a clear statement about methodology. It is not necessary to write the steps of the method, but briefly explain the method used, analysis, and tools used. Write the keywords in alphabetical order.

Response: The clear statement has been given, please see the new manuscript document.

2. In the initial paragraph in the introduction the problem is less clear from the author's point of view. It should refer after summarizing some of the results from the existing references. Some sentences that need to be corrected such as "...As a result, how to conduct innovative research on the space protection and vitality renewal of ancient towns with canals in the new historical period has become an important topic for the continuity and long-term development of those ancient towns in the modern era…” Further explanation is needed in this statement. The introduction is something that explains the contents of the entire manuscript. I suggest entering the contribution section in the introduction (contribution is part of introduction). Make the sentences flow and adjust the sentence by referring to the reference described in the initial paragraph in the introduction.

Response: The paragraph has been revised, please see line17-20.

3. Did you mean Yoshinobu Ashihara? (line 77).

Response: I mean in the ancient town, the original canal only providing water and aesthetics function, prefer to the limited function of original canal.

4. Literature review is a section that explains the basis of the study conducted. It is necessary to explain which parts of the reference are used in the methodology and then used to carry out the analysis. How the author(s) explains this into the methodology is important. It is highly recommended to summarize each part of the literature review by relating it to the research topic being carried out. State at the end of each paragraph each literature.

Response: This part has been revised, please see line90-91,104-105,125-127.

5. Paper for international journals will be read by readers who do not understand the location, so give information in the form of maps of Xixing, China, and which parts of China are studied.

Response: The new maps have been added, please see China map and Xixing ancient town location map.

6. Explain with a statement at the end of the discussion section that explains the analysis of the discussion that has been described. Therefore, it is necessary to distinguish which part of the results and which part of the discussion. Make it in its own part.

Response: The paragraph has been revised, distinguished the results, discussion and expectation of this study. Results was line 367-377.Discussion was 380-392. Expectation was 441-444.

7. Conclusion is the answer to the question asked in the introduction. So, the conclusion in this article should answer what is being questioned. Need to do a sharper explanation is actually what problems to be answered. In my opinion, there is no conclusion and it is too long for conclusion section, but it is also necessary to answer that should be at the end of the introduction. Please avoid writing conclusions in points, make in paragraph sentences. Two to three paragraphs for the conclusion section are sufficient.

Response: The structure of conclusions section has been adjusted, Please see line 428-432.

8. Each figure requires a caption and explanation in the image, for example adding text to the captured image calculation from DepthMap.

Response: All the figures in the manuscript have been advised ,please see the figure4-figure9.

9. In general, systematics in writing of scientific articles is Introduction, Literature review, Research Methods, Results and Discussion, and Conclusions. Systematics should be used in writing articles in this paper need to be improved.

Response: The systematics of the paper has been adjusted and improved accordingly.
