# Supplementary material for: Study on the sustainability of ancient canal towns on the basis of the topological structure analysis of streets and lanes: A case study of the Xixing ancient town
Source: PLoS One. 2023 Jan 6;18(1):e0279979. doi: 10.1371/journal.pone.0279979 (PMC9821790; doi:10.1371/journal.pone.0279979)
Supplement: Attachment — Submitted filename: PONE-D-22-18649_R2-review_comments.docx [file pone.0279979.s010.docx]

Dear Editor,

Thank you very much for offering me the opportunity to review this revised manuscript. My recommended minor corrections they can be firstly addressed, prior an “accepted as is” decision to be reached.

Thank you very much again. I am always at your reviewing disposal.

Kind regards,

Dr. Grigorios L. Kyriakopoulos

**Review comments for the revised manuscript: PONE-D-22-18649_R2**

*Study on the Sustainable Renewal Strategy of Ancient Towns with Canals on the Basis of the Topological Structure Analysis of Streets and Lanes: A Case Study of the Xixing Ancient Town*

The following minor corrections can be addressed prior to an “acceptance” decision.

- The manuscript title has to be shortened since it currently contains 31 words.
- There are extensive narrative points that are not cited, thus, checking and cross-citing them it is needed.
- It is not clear and straightforward what are the borders: start-end of the main sections: 1. Introduction, 2. Literature Review, 3. Methodology, 4. Results, 5. Discussion, 6. Conclusions and Future Research. Therefore, it is highly recommended:

-if main headings are missing to be added in the order above, while all main headings to be numbered as 1, 2, 3, …….., and their subheadings to be numbered as 2.2, 2.1, ……, 3.1, 3.2, ……., where missing and where matching to the narrative flow/text content.

-titles such as that of:

*“Local integration degree of the Xixing Ancient Town”*

*“The choice of the Xixing Ancient Town”*

*“Construction of the Adaptive Mode of the Integration of Living Environment and Business Space”*

To be shortened and placed as subheadings within the aforesaid main sections’ structure: 1,…., 6, where matching to the revised narrative flow.

- The concluding remarks (to be presented in a separated 6. Conclusions and Future Research section) they have to oriented towards the constraints, the limitations, and the future research orientations and propositions of generalized truth, beyond that of Xixing ancient town, of similar research, cultural and spatial contexts in China or worldwide, to be drawn. To this end 3-4 extra sentences can be added at this ending section 6.
- Special care and provision should be taken that all source citations from which the Figures and Tables have been developed/adapted/recalled from, to be explicitly noted in their legends/caption (where missing).
