# Supplementary material for: Study on the sustainability of ancient canal towns on the basis of the topological structure analysis of streets and lanes: A case study of the Xixing ancient town
Source: PLoS One. 2023 Jan 6;18(1):e0279979. doi: 10.1371/journal.pone.0279979 (PMC9821790; doi:10.1371/journal.pone.0279979)
Supplement: Attachment — Submitted filename: Response to Reviewers.docx [file pone.0279979.s011.docx]

Dear Editor,

We are truly grateful to yours and other reviewers’ comments on our manuscript (PONE-D-22-18649R2).

Based on these comments, we have made careful modifications on the original manuscript. The revised parts according to the comments are marked in blue, and other changes are in yellow. Below you will find our point-by-point responses to the reviewers’ comments/questions.

We hope that these revisions are satisfactory and that the revised version will be acceptable for publication in PLOS ONE journal.

Thank you very much for your work concerning our paper. Wish you all the best!

Weilu Lv, Ning Wang, Yun Huang

**Review comments for the revised manuscript: PONE-D-22-18649_R2**

*Study on the Sustainability of Ancient Canal Towns on the Basis of the Topological Structure Analysis of Streets and Lanes: A Case Study of the Xixing Ancient Town*

The following minor corrections can be addressed prior to an “acceptance” decision.

- The manuscript title has to be shortened since it currently contains 31 words.

Response: We have already shorten the manuscript title. The new title is: Study on the Sustainability of Ancient Canal Towns on the Basis of the Topological Structure Analysis of Streets and Lanes: A Case Study of the Xixing Ancient Town.

- There are extensive narrative points that are not cited, thus, checking and cross-citing them it is needed.

Response: We have checked the manuscript and cited the relevant points. Please see the new references.

- It is not clear and straightforward what are the borders: start-end of the main sections: 1. Introduction, 2. Literature Review, 3. Methodology, 4. Results, 5. Discussion, 6. Conclusions and Future Research. Therefore, it is highly recommended:

-if main headings are missing to be added in the order above, while all main headings to be numbered as 1, 2, 3, …….., and their subheadings to be numbered as 2.2, 2.1, ……, 3.1, 3.2, ……., where missing and where matching to the narrative flow/text content.

-titles such as that of:

*“Local integration degree of the Xixing Ancient Town”*

*“The choice of the Xixing Ancient Town”*

*“Construction of the Adaptive Mode of the Integration of Living Environment and Business Space”*

To be shortened and placed as subheadings within the aforesaid main sections’ structure: 1,…., 6, where matching to the revised narrative flow.

Response: We have numbered the heading titles of the main sections. Please see the new manuscript document.

- The concluding remarks (to be presented in a separated 6. Conclusions and Future Research section) they have to oriented towards the constraints, the limitations, and the future research orientations and propositions of generalized truth, beyond that of Xixing ancient town, of similar research, cultural and spatial contexts in China or worldwide, to be drawn. To this end 3-4 extra sentences can be added at this ending section 6.

Response: We have summarized the limitations and proposed two directions for future research to broaden the research perspective and deepen research thinking. Please see the 7. Expectations section.

- Special care and provision should be taken that all source citations from which the Figures and Tables have been developed/adapted/recalled from, to be explicitly noted in their legends/caption (where missing).

Response: We have marked the sources of the Figures and Tables. Please see the new manuscript document.
