# Supplementary material for: Study on the sustainability of ancient canal towns on the basis of the topological structure analysis of streets and lanes: A case study of the Xixing ancient town
Source: PLoS One. 2023 Jan 6;18(1):e0279979. doi: 10.1371/journal.pone.0279979 (PMC9821790; doi:10.1371/journal.pone.0279979)
Supplement: Attachment — Submitted filename: Response to Reviewers.docx [file pone.0279979.s012.docx]

Dear Editor,

We are truly grateful to the comments on our manuscript (PONE-D-22-18649R2).

Based on the comments, we have made careful modifications on the original manuscript. The revised parts according to the comments are marked in blue. Below you will find our point-by-point responses to the reviewers’ comments/questions.

We hope that these revisions are satisfactory and that the revised version will be acceptable for publication in PLOS ONE journal.

Thank you very much for your work concerning our paper. Wish you all the best!

Weilu Lv, Ning Wang, Yun Huang

**Review comments for the revised manuscript: PONE-D-22-18649_R2**

*Study on the Sustainability of Ancient Canal Towns on the Basis of the Topological Structure Analysis of Streets and Lanes: A Case Study of the Xixing Ancient Town*

- Reviewer #1: This paper discusses the spatial syntax to analyze the topological structure of roads and alleys in the ancient city of Xixing in the Zhedong canal as a case object. I think this topic is interesting regard to most situations in urban development. The author has revised most of what has been suggested. But there are minor revisions that must be corrected.
  In this version of the article, the conclusion section is omitted. The author must write a conclusion, as stated in the previous revised version "Conclusion and Expectations", “…Based on the theory and method of spatial syntax, this paper studied the spatial and topological configuration…” In this version it is even removed. Stick to this conclusion statement. Please rewrite the section as in the previous version.

Response: We have already added the conclusion at the last section. Please see the 7. Conclusion and Expectations section.

- Reviewer #3: At this revised manuscript authors improved their analysis and findings into a satisfactory and meaningful manner. In this respect the revised manuscript sustains novel features and it can be accepted for publication at the PLOS ONE journal as is.

Response: Thanks a lot!
